# Activation of the steroid and xenobiotic receptor, SXR, induces apoptosis in breast cancer cells

**DOI:** 10.1186/1471-2407-9-3

**Published:** 2009-01-05

**Authors:** Suman Verma, Michelle M Tabb, Bruce Blumberg

**Affiliations:** 1 Department of Developmental and Cell Biology, 5205 McGaugh Hall, University of California, Irvine, CA 92697-2300, USA

## Abstract

**Background:**

The steroid and xenobiotic receptor, SXR, is an orphan nuclear receptor that regulates metabolism of diverse dietary, endobiotic, and xenobiotic compounds. SXR is expressed at high levels in the liver and intestine, and at lower levels in breast and other tissues where its function was unknown. Since many breast cancer preventive and therapeutic compounds are SXR activators, we hypothesized that some beneficial effects of these compounds are mediated through SXR.

**Methods:**

To test this hypothesis, we measured proliferation of breast cancer cells in response to SXR activators and evaluated consequent changes in the expression of genes critical for proliferation and cell-cycle control using quantitative RT-PCR and western blotting. Results were confirmed using siRNA-mediated gene knockdown. Statistical analysis was by t-test or ANOVA and a P value ≤ 0.05 was considered to be significant.

**Results:**

Many structurally and functionally distinct SXR activators inhibited the proliferation of MCF-7 and ZR-75-1 breast cancer cells by inducing cell cycle arrest at the G1/S phase followed by apoptosis. Decreased growth in response to SXR activation was associated with stabilization of p53 and up-regulation of cell cycle regulatory and pro-apoptotic genes such as p21, PUMA and BAX. These gene expression changes were preceded by an increase in inducible nitric oxide synthase and nitric oxide in these cells. Inhibition of iNOS blocked the induction of p53. p53 knockdown inhibited up-regulation of p21 and BAX. We infer that NO is required for p53 induction and that p53 is required for up-regulation of cell cycle regulatory and apoptotic genes in this system. SXR activator-induced increases in iNOS levels were inhibited by siRNA-mediated knockdown of SXR, indicating that SXR activation is necessary for subsequent regulation of iNOS expression.

**Conclusion:**

We conclude that activation of SXR is anti-proliferative in p53 wild type breast cancer cells and that this effect is mechanistically dependent upon the local production of NO and NO-dependent up-regulation of p53. These findings reveal a novel biological function for SXR and suggest that a subset of SXR activators may function as effective therapeutic and chemo-preventative agents for certain types of breast cancers.

## Background

Anti-estrogens such as tamoxifen are important therapeutic agents in the treatment and chemoprevention of breast cancer [1]. Other compounds such as fatty acid amides and retinoid × receptor (RXR) agonists are also effective against breast cancer in cell lines and in animal models [2,3]. Interestingly, a macrolide antibiotic-rifampicin, the antimycotic drug clotrimazole, endogenous cannabinoids such as anandamide, RXR agonists (rexinoids) such as targretin, and tocotrienol forms of Vitamin E share the ability to inhibit the growth of various types of cancer [2-8]. Some of these compounds such as rifampicin, targretin, and tocotrienols have also been shown to have synergistic or additive effects against cancer when used together with tamoxifen [4,7,9]. Although it is certainly possible that they act through separate and distinct pathways, the ability of these structurally and functionally distinct compounds to inhibit the growth of cancer cells and their additive effects with tamoxifen raise the possibility that they might also act through a common molecular target. Notably, these compounds (including tamoxifen) share the ability to activate a heterodimer of the steroid and xenobiotic receptor (SXR [10], also known as PXR [11], PAR [12], and NR1I2 [13]) and retinoid × receptor (RXR) [14].

SXR is an orphan nuclear receptor activated by a large number of endogenous steroids and bile acids, prescription drugs, dietary compounds and xenobiotic compounds [15-18]. SXR is highly expressed in the liver and intestine where it acts as a broad-specificity chemical sensor. SXR activation induces expression of genes involved in all three phases of drug and xenobiotic metabolism: hydroxylation by cytochrome P450 enzymes, conjugation to glutathione, sulfates and sugars, and transport by ABC family transporters (reviewed in [19-21]). We and others have shown that SXR is also expressed in tissues such as bone, kidney, lung, endometrium, and breast [22-26], where it may play roles other than its conventional role in metabolism. For example, activation of SXR in bone is associated with increased expression of bone biomarkers involved in maintaining bone homeostasis [27,28]. SXR activation in liver is associated with decreased fibrogenesis and increased expression of anti-apoptotic genes such as BCL2 and BCL-XL [29-31]. Expression of SXR in endometrial cancer tissues is associated with decreased sensitivity to anti-cancer agents [22,32]. Recently, one report has shown that activation of SXR leads to heightened sensitivity to oxidative cellular damage and apoptosis in transgenic mice and in cancer cells [33]. Another suggested an anti-apoptotic role for SXR in colon cancer cells and in normal mouse colon epithelium [34]

SXR is expressed in breast cancer cells and tissues [23,25]. The expression of SXR in breast cancer cells and tissues and the ability of numerous compounds active against breast cancer to activate SXR led us to hypothesize that SXR might serve as a common molecular target for their action. Here we show that SXR activation can inhibit breast cancer cell growth by inducing cell cycle arrest and apoptosis. We define a molecular pathway wherein activation of SXR inhibits proliferation of estrogen receptor positive (ER^+^) and p53 wild type (p53^wt^) breast cancer cell lines (MCF-7 and ZR-75-1) via induction of inducible nitric oxide synthase (iNOS), increased expression of nitric oxide (NO), and NO-dependent stabilization and accumulation of p53. SXR-mediated stabilization of p53 protein and up-regulation of p53 mRNA leads to increased expression of p53-dependent cell cycle regulators and pro-apoptotic genes such as p21, BAX and PUMA. Gain- and loss-of-function studies confirm that SXR is the mediator of this pathway. We conclude that activation of SXR is anti-proliferative in MCF-7 and ZR-75-1 breast cancer cells and that these effects are mediated through a NO and p53-dependent pathway. Our results identify a novel molecular pathway for SXR action in breast cancer and widen the biological relevance of SXR beyond xenobiotic metabolism.

## Methods

### Cell culture

Rifampicin, anandamide and camptothecin were from BioMol (Plymouth Meeting, PA) and all other compounds were from Sigma. Compounds were freshly diluted in DMSO prior to addition to cell culture media. The final DMSO concentration was < 0.05%. Breast cancer cell lines were maintained in phenol red free Improved Minimum Essential Media (IMEM; Mediatech, Kansas City, MO) supplemented with 10% fetal bovine serum (FBS; Invitrogen Corporation) (IMEM/FBS). LS180 cells were maintained in Dulbecco Modified Eagle Medium (DMEM; Cellgro) supplemented with 10% FBS.

### Quantitative real-time RT-PCR

Cell lines were treated with SXR ligands for the indicated times followed by isolation of total cellular RNA using Trizol reagent (Invitrogen). After removal of potentially contaminating genomic DNA by DNAse digestion and LiCl precipitation, 1 μg total RNA was reverse transcribed using Superscript III (Invitrogen) following the manufacturer's recommended protocol. Quantitative real time RT-PCR (QRT-PCR) was performed using primer sets as shown in Table 1 using SYBR Green PCR Master Mix (Applied Biosystems, Foster City, CA) or FastStart SYBR Green Master Mix (Roche Applied Science, Indianapolis, USA) in a DNA Engine Opticon-Continuous Fluorescence Detection System (MJ Research, Reno, NV). All samples were quantitated by the comparative cycle threshold C(t) method for relative quantitation of gene expression, normalized to either GAPDH or β-actin [35].

**Table 1:** Primer sequences used in Q-RTPCR analysis

Name	Forward	Reverse
SXR	AGGATGGCAGTGTCTGGAAC	AGGGAGATCTGGTCCTCGAT
iNOS	CACCATCCTGGTGGAACTCT	TCCAGGATACCTTGGACCAG
eNOS	GTTACCAGCTAGCCAAAGTC	GACAGGAAATAGTTGACCATCTC
nNOS	GAAGAAAGCAACCAGAGTCAG	GTCCAAATCTCTGTCCACCT
p53	CCGCAGTCAGATCCTAGC	AATCATCCATTGCTTGGGACG
p21	GCGATGGAACTTCGACTTTG	CAGGTCCACATGGTCTTCCT
BAX	GGGGACGAACTGGACAGTAA	CAGTTGAAGTTGCCGTCAGA
PUMA	GACCTCAACGCACAGTACGA	CTAATTGGGCTCCATCTCG
CYP3A4	GGCTTCATCCAATGGACTGCA TAAAT	TCCCAAGTATAACACTCTACACAGACAA
Calmodulin	TTGACTTCCCCGAATTTTTGACT	CTGCACTGATATAACCATTGCCA
β-actin	GGACTTCGAGCAAGAGATGG	AGGAAGGAAGGCTGGAAGAG
GAPDH	GGCCTCCAAGGAGTAA	AGGGGAGATTCAGTGTGGTG

### Western blotting

Cells growing in culture were washed three times with ice-cold PBS and then lysed in RIPA buffer (137 mM NaCl, 20 mM Tris-HCl, pH 7.5, 1% Triton X-100, 0.5% NP-40, 10% glycerol, 2 mM EDTA, pH 8.0) plus protease inhibitors (Roche). Lysate protein concentrations were determined using the Bradford method and equal amounts of protein were separated on 8–10% SDS-polyacrylamide gels. For CaM protein, the proteins were separated on 4–20% gradient Tris-HCl gels. Proteins were transferred to Immobilon membrane (Millipore) using the semi-dry method (BioRad, Hercules, CA). Membranes were blocked for 1 hr with 5% non-fat dried milk in TBST (25 mM Tris-HCl pH 7.4, 135 mM NaCl, 2.5 mM KCl, 0.1% Tween-20) and then incubated overnight at 4°C with SXR (Anti-412, or PP-H4417, Perseus Proteomics inc., Japan) or p53 (FL-393 HRP, Santa Cruz Biotechnology Inc., USA) antibodies. The membranes were fixed in 0.2% glutaraldehyde in TBS for CaM protein before blocking in 3% BSA overnight followed by 1 hr incubation in mouse monoclonal CaM antibody (C-7055, Sigma-Aldrich, USA) at RT. The anti-412 SXR antibody was raised against a synthetic peptide sequence starting at amino acid 412 (CLRIQDIHPFATPLMQE), by Bethyl Laboratories, Inc., Montgomery, TX. Isolated IgG was affinity purified against this peptide prior to use. The primary antibody incubation was followed by 1 hr incubation at room temperature with HRP-conjugated secondary antibody (1:10,000; Santa Cruz Biotechnology Inc., USA). The bands were detected using the ECL Plus Western Blotting Detection System (Amersham Bioscience, USA). Chemiluminescence was assayed using an Alpha Innotech Fluorchem SP imager (Alpha Innotech Inc., CA, USA) and analyzed by densitometry using FluorChem AlphaEase FC software (Alpha Innotech).

### Proliferation assays

MCF-7 cells were seeded at 500 cells/well and ZR-75-1 cells were seeded at 2500 cells/well in 96-well plates in IMEM supplemented with 10% FBS. Cells were treated with ligands at the indicated concentrations every other day for seven days. Cell proliferation was measured using a fluorescence assay (CyQuant, Molecular Probes) and a Cytofluor 4000 Fluorescence Multi-Well Plate Reader (PerSeptive Biosystems).

### Flow Cytometry

MCF-7 cells were incubated with 10 μM of SXR agonists (rifampicin, tamoxifen, anandamide, clotrimazole, RU486) or DMSO solvent control for 24 h. Cells were subsequently harvested using trypsin (0.5% w/v), centrifuged (140 *g *for 12 min), washed with PBS, and fixed with 2 ml ice-cold 70% ethanol overnight. Cells were centrifuged and resuspended with 10 μg/ml Propidium Iodide and 100 μg/ml RNase A at 37°C for 30 min. Samples were acquired on a FACSCalibur (BD Biosciences), and data were analyzed by CellQuest (BD Biosciences) and FlowJo (Tree Star, San Carlos, CA) software. Modfit (Verity Software, Topsham, ME) software was used to quantitate cell cycle using the fluorescence values of the FL2-area channel.

### Detection of apoptosis

MCF-7 cells growing in culture were treated with 10 μM SXR ligands for 24, 48 and 72 hrs. Cells were also treated with 10 μM camptothecin for 24 hrs as a positive control for apoptosis. After the indicated period of treatment, cells were trypsinized and counted. Apoptosis was measured in the cytoplasmic fraction of equal numbers of cells using the Cell Death Detection ELISA (Roche Applied Science, Germany) using the manufacturer's recommended protocol.

### NOS activity in cell lysates

MCF-7 cells were treated with 10 μM rifampicin or DMSO for indicated time periods. The cells were also treated with a cocktail of IL-1β (20 ng/ml), TNFα (15 ng/ml) and LPS (1 mg/ml) as a positive control for iNOS induction. After the indicated time periods, cell lysates were made in 1× homogenization buffer (25 mM Tris-Hcl pH7.4, 1 mM EDTA, 1 mM EGTA) by sonicating cells twice for 10 s at 10 amp. NOS activity was detected by measuring the conversion of ^14^C L-arginine (Amersham Bioscience, USA) into ^14^C L-citrulline by using nitric oxide synthase assay kit (Calbiochem Inc., USA). Radioactivity was measured by liquid scintillation counting and normalized to protein content.

### Nitrite concentration in culture medium

NO production was measured as nitrite concentration in cell culture supernatants using the Griess method [36]. Briefly, aliquots of media were removed from cells growing in culture in the presence or absence of SXR activators, followed by centrifugation to remove cells. In the cell-free supernatants nitrate, a stable metabolite of NO, was reduced to nitrite by incubating sample aliquots for 15 min at 37°C in the presence of 0.1 U/ml nitrate reductase (from *Aspergillus *species, Roche), 50 μM NADPH (Sigma, USA) and 5 μM FAD (Sigma, USA). When nitrate reduction was complete, NADPH was oxidized to avoid interference with subsequent nitrite determination. For this purpose, the reduced samples were incubated with 10 U/ml L-lactate dehydrogenase (from rabbit muscle) and 10 mM sodium pyruvate for 5 min at 37°C, followed by addition of 50 μl of 1% sulfanilic acid in 5% phosphoric acid and 50 μl of 0.1% N-(1-naphthyl) ethylenediamine dihydrochloride to 100 μl of the reduced cell-free supernatant [36]. After 10 minutes at 23°C, the absorbance at 546 nM was determined. Concentrations of nitrite in samples were calculated from standard curves using sodium nitrite as the reference compound.

### RNA Interference

Small interfering RNA (siRNA) duplexes targeting human SXR and p53 were custom synthesized by Qiagen (Qiagen Inc. USA). The most effective SXR siRNA target sequence was 5'-GGCCACTGGCTATCACTTC-3' [27,37] and p53 siRNA sequence was 5'-AAACCACTGGATGGAGAATATTT-3'. Non-silencing control siRNA sequence 5'-AATTCTCCGAACGTGTCACGT-3' (Qiagen Inc. USA) was used as a negative control. The cells were transfected using Lipofectamine™ RNAiMAX transfection reagent (Invitrogen Inc., USA). The knock down efficiency by mRNA and protein were determined after 48 and 72 hrs of transfection, respectively. For gene expression assays, the cells were transfected with siRNA two day prior to ligand treatment, or the day of ligand treatment. After 48 hr of treatment with SXR ligands, RNA was isolated, cDNA synthesized and QRT-PCR analysis performed.

### Statistical analyses

Data are shown as the mean values ± SEM. Results of two groups were analyzed using "unpaired" Student's *t *tests. Multiple comparisons were analyzed using one-way analysis of variance (ANOVAs). *P *≤ 0.05 was taken to be statistically significant.

## Results

### SXR mRNA and protein are expressed in breast cancer cell lines

**Figure 1:**
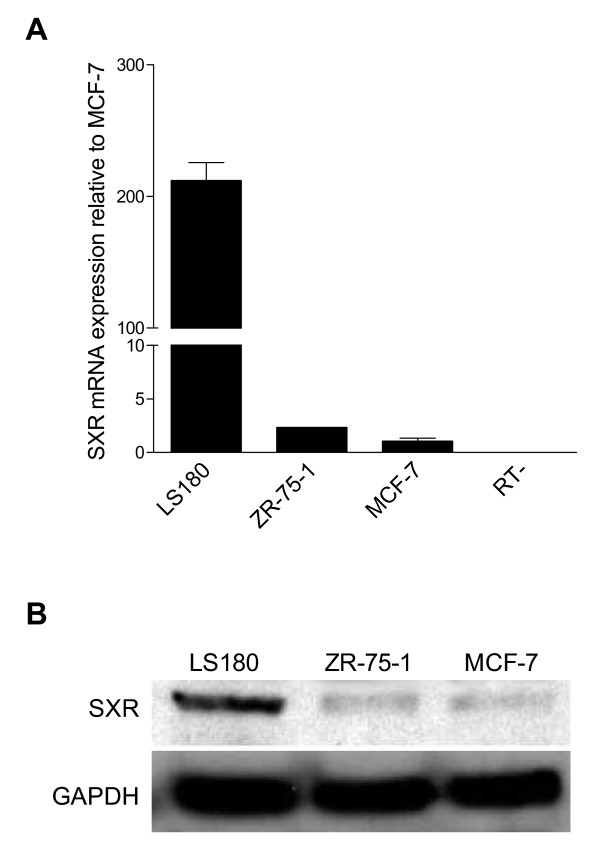
**SXR mRNA and protein are expressed in breast cancer cell lines**. A) Total RNAs from breast cancer cell lines, MCF-7 and ZR-75-1 and colon carcinoma cell line LS180 were isolated, reverse transcribed and subjected to QRT-PCR analysis using primers for human SXR. Values represent the average of triplicates ± S.E.M. and were replicated in at least three independent experiments. B) Equal amounts of total cell lysates (30 μg-in each lane) were separated by SDS-PAGE followed by Western blot analysis using the rabbit polyclonal anti-SXR (anti-412) antibody. The blot was stripped and re-probed with anti-GAPDH antibody (mouse monoclonal 6C5, Ambion, Inc., Austin, TX) as a loading control. The results were replicated in at least three independent runs.

A variety of different classes of compounds that are able to transcriptionally activate SXR (e.g., anandamide, rexinoids, tamoxifen) have been previously observed to slow the proliferation of breast cancer cell lines and/or slow tumor growth in rodent models of breast cancer [3,4,7,38]. Previous work and our preliminary results showed that SXR was expressed in human breast cancers and in some breast cancer cell lines [23,25]. Therefore, we hypothesized that SXR activation might mediate some of the anti-proliferative effects of these compounds in breast cancer. We first surveyed the expression of SXR in two established breast cancer cell lines, MCF-7 and ZR-75-1. SXR mRNA was present in both cell lines and the level of SXR mRNA was lower in the breast cancer cells than in the positive control colon carcinoma cell line LS180 (Figure 1A). However, SXR expression is known to be high in the liver and gut where it regulates the xenobiotic response [10,39].

Considering the relatively low levels of SXR mRNA found in breast cancer cells, western blot analysis of cell lysates was employed to confirm that SXR protein was, in fact, expressed. In agreement with the QRT-PCR data, SXR protein was present in both breast cancer cell lines (for characterization of the antibody, see Additional file 1: supplementary Figure 1). Consistent with these findings, MCF-7 and ZR-75-1 cells treated with 10 μM of the SXR activators rifampicin, anandamide or clotrimazole for 48 hours showed induction of the well-characterized SXR target gene CYP3A4 by QRT-PCR, demonstrating the functionality of endogenous SXR in these cells (data not shown). The kinetics of CYP3A4 up-regulation (48 hour post-treatment) by SXR activators matches with previously reported timing for its induction by SXR activators in osteosarcoma cells [24], where its activation induces genes involved in bone homeostasis.

### SXR activation reduces the proliferation of breast cancer cell lines

Next, to test our hypothesis that SXR might play a role in some of the anti-proliferative effects previously observed by different classes of compounds (e.g., anandamide, rexinoids, tamoxifen); we did an initial screening experiment in which we tested a large number of structurally and functionally diverse compounds that all share the ability to activate SXR. We hypothesized that similar effects elicited by these distinct compounds could be through a common, SXR-mediated mechanism. For this purpose, the breast cancer cell lines, MCF-7 and ZR-75-1 were treated with compounds such as macrolide antibiotic rifampicin; which is a human selective SXR activator [10], the anti-estrogen tamoxifen; which is also an SXR agonist [14,40], the fatty acid ethanolamide anandamide, the anti-fungal clotrimazole [41] or the calcium channel blocker nifedipine [42]. Prior to treatment, the ability of these compounds to activate SXR was confirmed in transient transfection assays (see Additional file 1: supplementary Figure 2A). The phtoestrogen-rutin did not activate SXR in transfection assays so that was chosen as a negative control for the experiment.

**Figure 2:**
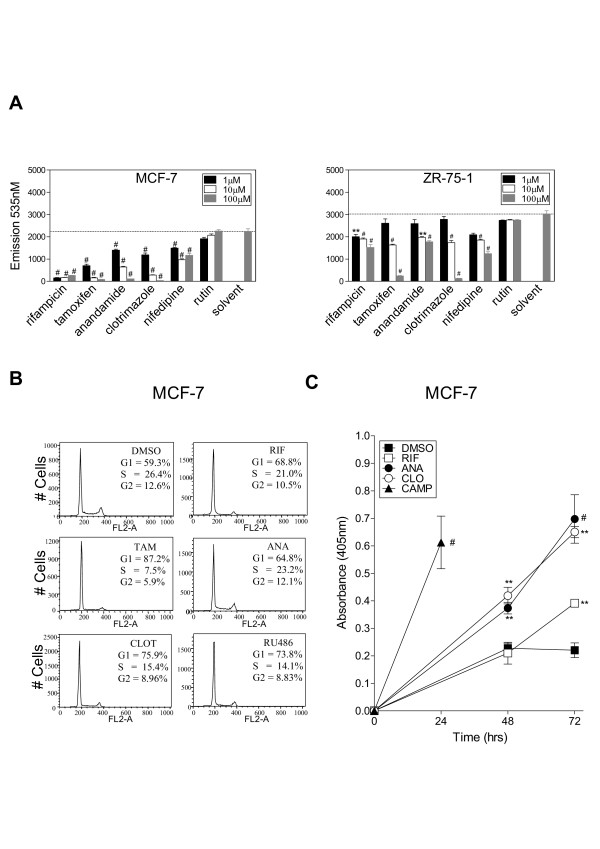
**SXR activation reduces proliferation of breast cancer cells**. A) MCF-7 and ZR-75-1 cells were grown in the presence of 1, 10 or 100 μM concentrations of SXR activators. Rutin, an isoflavone that does not activate SXR, and DMSO solvent were used as negative controls. Cell proliferation was measured after seven days using a fluorescence assay wherein emission at 535 nm is proportional to cell number. The values represent the average emission for triplicates ± S.E.M. These results were replicated in independent experiments. ** represents P ≤ *.01 and # represents P *≤ *.001 *in comparison to solvent control (by one way ANOVA analysis) B) SXR activators cause G0/G1 arrest as compared to DMSO solvent controls. MCF-7 cells treated with 10 μM SXR activators for 24 hours were fixed, stained with propidium iodide and data acquired on FACSCalibur (BD Biosciences). Data were analyzed by CellQuest (BD Biosciences) and FlowJo (Tree Star, San Carlos, CA) software. Modfit (Verity Software, Topsham, ME) software was used to quantitate cell cycle using the fluorescence values of the FL2-area channel. The experiment was repeated twice in duplicates and the numbers represent the average of four values. Note: to measure the cell cycle distribution the population was live gated. C) SXR activators cause apoptosis in MCF-7 cells starting at 48 hours post-treatment. MCF-7 cells were grown in the presence of 10 μM SXR activators rifampicin, anandamide, or clotrimazole (RIF, ANA or CLO). Apoptosis was measured at 24, 48 and 72 h in the cytoplasmic fraction using the Cell Death Detection ELISA (Roche Applied Science, Germany). DMSO treatment was used as a negative control and 24 hour camptothecin (CAMP) treatment was used as a positive control for apoptosis. The values represent average of triplicates ± S.E.M and the results were replicated in two independent experiments. ** represents P ≤ *.01 and # represents P *≤ *.001 *(by one way ANOVA analysis).

To determine the dose dependent effect of SXR activators on proliferation, breast cancer cells were cultured in the presence of a concentration series (1, 10 and 100 μM) of test compounds or controls (solvent, rutin). To measure the integrated effect on cell cycle and/or cell survival in the presence of SXR activators the proliferation assays were continued until 7 days which was the time when the cells in the solvent control wells just reached confluency. Interestingly, SXR activators elicited a dose-dependent reduction in proliferation of both cell types (Figure 2A). The rank order of potency in the proliferation assay agreed well with the potency and efficacy of these compounds as SXR activators. For example, rifampicin, which was the best activator of SXR in transfection assays, also had the highest ability to inhibit proliferation of breast cancer cells in proliferation assay. Rutin, which did not activate SXR in transfection assays, lacked any effect on proliferation of cancer cells (Figure 2A and Additional file 1: supplementary Figure 2). The anti-proliferative effects of tamoxifen were more pronounced than its ability to activate SXR, but, as both of the tested breast cancer cell lines are estrogen receptor positive, it is likely that some of the observed effects are mediated through an ER-dependent mechanism.

### SXR activation induces apoptosis and cell cycle arrest in breast cancer cells

The decreased proliferation observed in breast cancer cell lines treated with SXR activators could be due to inhibition of proliferation, increased cell death or both. To determine the earlier molecular events responsible for the ultimate effect on cell number, we first examined the effects of SXR activator treatment on the cell cycle using flow cytometry. In proliferation assays, the growth inhibitory effects of SXR activators were obvious at doses as low as 10 μM (Figure 2A). Therefore, we chose a 10 μM dose of SXR activator compounds for all further experiments. MCF-7 cells were treated with SXR activators rifampicin, tamoxifen, anandamide, clotrimazole, RU486 [41] or solvent control for a time course of 12, 24 and 48 hrs so that the earlier molecular events can be detected within one or two cell cycles. Cellular DNA content was measured by propidium iodide staining followed by FACS analysis. All SXR activator treated MCF-7 cells exhibited a consistent increase in the fraction of cells in the G0/G1 phase of the cell cycle compared with controls starting at 24 hours, suggesting that they are arrested at G0/G1 (Figure 2B). The cells treated with SXR activators accumulated more in G1/S phase at 48 hr but the cells in solvent control also increased in G1/S phase at 48 hr time point, suggesting that the solvent control cells have started to get confluent at that time. The SXR activators consistently had more cells in G1/S phase than did solvent controls, suggesting that SXR activators can specifically cause G1/S arrest in breast cancer cells (data not shown).

This finding is consistent with published results showing that tamoxifen (an anti-estrogen and SXR activator) causes G0/G1 arrest in MCF-7 cells [43]. Although tamoxifen, RU486 and nifedepine can activate SXR, they can also affect ER and PR/GR and calcium homeostasis of the cells respectively, which can have erroneous effects on cell growth. To avoid unnecessarily confounding the results, we decided to use only the antibiotic rifampicin, the anti-fungal clotrimazole and the fatty acid amide anandamide as SXR activators in subsequent experiments.

We next tested the effect of SXR activation on apoptosis using a sandwich ELISA with anti-histone and anti-DNA antibodies. Again MCF-7 cells were treated with 10 μM of the SXR activators rifampicin, anandamide, clotrimazole or solvent from 24–72 hours, or treated with 10 μM camptothecin for 24 hours as a positive control for induction of apoptosis. Each of the SXR activators increased the amount of apoptosis observed in treated MCF-7 cells beginning at 48 hours (Figure 2C). Therefore, we infer that the decreased proliferation observed in MCF-7 cells is caused by an increase in cell cycle arrest at G0/G1 phase followed by apoptosis.

### Treatment with SXR activators increases expression of p53 and p53 target genes

**Figure 3:**
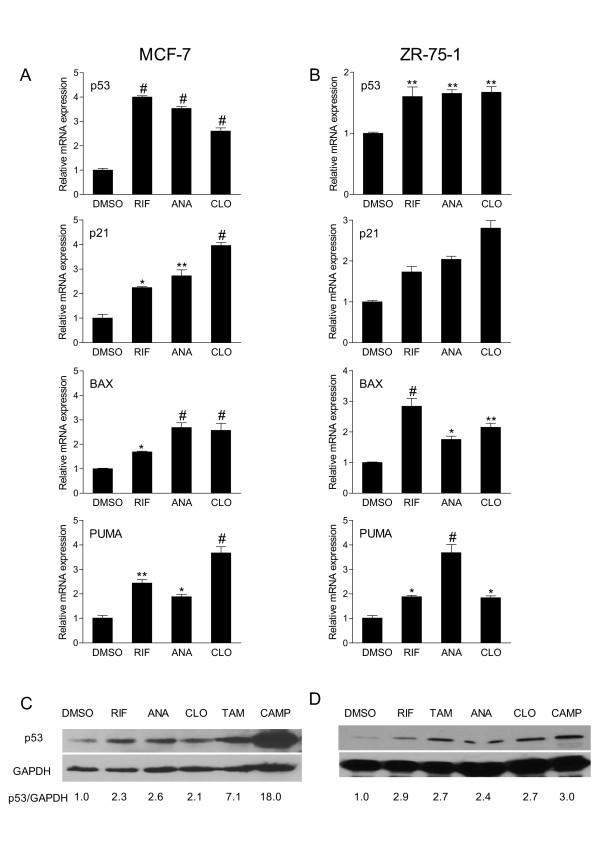
**Treatment with SXR activators increases expression of p53 and p53 target genes**. Total RNA was isolated from A) MCF-7 cells or B) ZR75-1 cells grown in the presence of 10 μM SXR activators rifampicin, anandamide, or clotrimazole (RIF, ANA or CLO) for 72 hours and 24 hours respectively. RNA was reverse transcribed and analyzed by QRT-PCR using primers for human p53, p21, BAX and PUMA. Data are depicted as average fold induction relative to solvent control in triplicates ± S.E.M. These results were replicated in at least three independent experiments. *represents P ≤ .05, **represents P ≤ *.01 and # represents P *≤ *.001 *(by one-way ANOVA analysis). C) SXR activation causes p53 accumulation. Cell lysates made from C) MCF-7 and D) ZR-75-1 cells treated with SXR agonists (10 μM) or solvent controls for 72 and 24 hours respectively were subjected to Western blot analysis using p53 antibody (FL-393 HRP, Santa Cruz Inc.). Equal loading was confirmed by stripping and re-probing the same blot with an anti-GAPDH antibody. Camptothecin (CAMP) 24 hour treatment was used as a positive control for p53 induction. The chemiluminescent bands were acquired by Alpha Innotech Fluorchem SP imager (Alpha Innotech Inc., CA, USA) and analyzed by spot densitometry analysis using FluorChem AlphaEase FC software (Alpha Innotech). The experiment was done in duplicates and the numbers represent the average from two independent runs. Note that the gap in ANA lane of ZR-75-1 is because of gel tearing at the time of transferring.

SXR is a transcription factor; therefore, we next looked for gene expression changes that resulted from the activation of endogenous SXR that could ultimately result in either apoptosis or cell cycle arrest. MCF-7 and ZR-75-1 cells were treated with 10 μM rifampicin, anandamide, clotrimazole or solvent controls, and total RNA was prepared after 24, 48 and 72 hours of incubation. The expression of a panel of genes involved in apoptosis, control of cell proliferation and cell cycle regulation were analyzed by QRT-PCR. Interestingly, we found that expression of mRNAs encoding p53 and three p53 target genes, p21, BAX and PUMA [44], were increased by all three SXR activators in both MCF-7 and ZR-75-1 cells. The changes in gene expression were statistically significant by all three test compounds by 72 hours in MCF-7 cells and by 24 hours in ZR-75-1 cells (Figure 3A and Figure 3B). The p53 expression went down by 72 hrs in response to all three test compounds in ZR-75-1 cells (data not shown). Increased expression of p21 induces cell cycle arrest or apoptosis [45], whereas BAX and PUMA are promoters of apoptosis [46-48]. Therefore, increased expression of BAX and PUMA is consistent with the apoptosis observed. Increased p21 is consistent with the G1 arrest observed in MCF-7 cells. These molecular changes in both MCF-7 and ZR-75-1 cells by all three test compounds led us to infer that SXR can increase expression of p53 and several key p53 target genes associated with apoptosis and inhibition of cell proliferation in breast cancer cell lines.

We also tested the effects of SXR agonists on steady state levels of p53 protein in MCF-7 and ZR-75-1 cells. Camptothecin treatment for 24 hrs served as a positive control for p53 induction. Treatment with each of the SXR activators resulted in increased p53 protein levels in both MCF-7 (Figure 3C) and ZR-75-1 (Figure 3D) cells. Previous reports showed that iNOS-mediated increases in nitric oxide (NO) levels lead to p53 stabilization and activation as a result of phosphorylation of p53 at ser-15 [49]. This phospho-p53 is transcriptionally active and increases expression of p21 and BAX [50,51]. Since iNOS is a reported target gene of ligand-activated SXR [52], we further investigated the idea that the mechanism linking SXR activation with the observed effects on p53 and its target genes involved iNOS expression and increased NO levels.

### Inducible nitric oxide synthase expression and nitric oxide levels are increased by SXR activation

**Figure 4:**
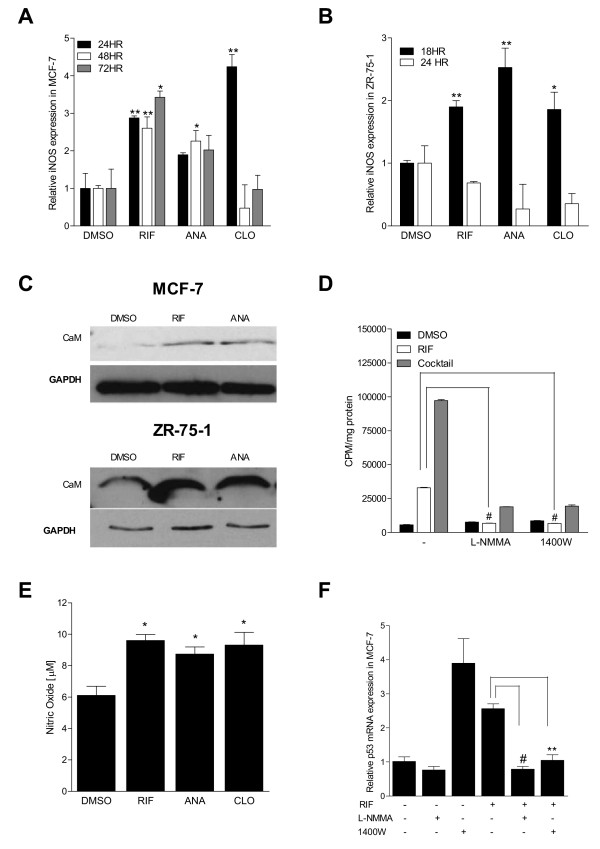
**SXR activation increases expression of inducible nitric oxide synthase and raises NOS activity.** A) MCF-7 and B) ZR-75-1 cells were grown in the presence of 10 μM concentration of SXR activators compounds rifampicin, anandamide or clotrimazole for 24, 48 and 72 hours and for 18 and 24 hours respectively. After the indicated time of treatment, total RNA was isolated, reverse transcribed and analyzed by QRT-PCR using primers for the human iNOS gene. Data are shown as average fold induction relative to solvent control in triplicates ± S.E.M. Results were replicated in at least three independent experiments.*represents P ≤ *.05 *and ** represents P ≤ *.01 *compared to control (by student's t test) C) SXR activation increase the levels of calmodulin protein in MCF-7 and ZR-75-1 cells. Equal amount of total cell lysates made from MCF-7 and ZR-75-1 cells treated with SXR activator compounds or solvent control for 24 and 18 hrs respectively were subjected to Western blot analysis using CaM antibody (C7055, sigma-aldrich) as described in materials and methods section. Equal loading was confirmed by probing with anti-GAPDH antibody. D) SXR activation increases iNOS activity. MCF-7 cells were activated by rifampicin or solvent control for 48 hours. Cytokine cocktail (IL1β (20 ng/ml), TNFα (15 ng/ml) and LPS(1 mg/ml) was used as a positive control. Nitric oxide synthase activity was determined intotal cells homogenates in the presence or absence of NOS inhibitors; L-NMMA and 1400 W (10 μM) as described in Materials and Methods. The data are depicted as counts per minute (CPM) per mg protein. The results have been replicated in independent runs. *# represents P *≤ *.001 *in comparison to RIF (by student's t test) E) SXR activation leads to Nitric Oxide (NO) accumulation. MCF-7 cells were treated with SXR agonists (10 μM) or solvent control for 48 hours. The concentration of nitrate plus nitrite (stable metabolites of nitric oxide) in the culture supernatant of SXR agonists treated or control-treated MCF-7 cells was measured using the Griess method [36]. Data are depicted as average fold induction relative to solvent control in triplicates ± S.E.M. The results were replicated in at least three independent experiments. *represents P ≤ *.05 *compared to control (by one-way ANOVA analysis). F) L-NMMA and 1400 W, the nitric oxide synthase inhibitors block the increase in expression of p53 caused by rifampicin. MCF-7 cells were pre-treated with or without L-NMMA (500 μM) or 1400 W (10 μM) for 1 hr and then stimulated by rifampicin for 72 hours. Total RNA was isolated from the cells after completion of treatment and analyzed for p53 expression using QRT-PCR. The data are shown as average fold induction relative to solvent control in triplicates ± S.E.M. rifampicin, RIF; tamoxifen, TAM; anandamide, ANA; clotrimazole, CLO; camptothecin, CAMP. *# represents P *≤ *.001 *and ** *represents *P ≤ .01 in comparison to RIF (by student's t test). rifampicin, RIF; anandamide, ANA; clotrimazole, CLO.

Although p53 mRNA expression is increased by treatment with SXR activators, the time course of induction in mRNAs encoding p53 and its target genes such as p21, BAX and PUMA (72 hours post-treatment in MCF-7 cells, and 24 hours post-treatment in ZR-75-1 cells) led us to hypothesize that elevation of p53 mRNA levels and the resulting changes in p53 target gene expression were a secondary response to an earlier primary effect of SXR activation. The p53 tumor suppressor can be activated or stabilized in response to DNA damage or cellular stress such as accumulation of reactive oxygen species (ROS) or reactive nitrogen species (RNS) [53]. Moreover, RNS-stabilized p53 has been shown to up-regulate both p21 and BAX expression [50,51]. Nitric oxide itself increases expression of p21 and causes late G1 arrest in cells through p53-mediated and p53-independent pathways [50,54]. Therefore, we considered the possibility that treatment with the various SXR activators was increasing the expression of a cellular stressor such as RNS. A previous report suggested that the promoter of inducible nitric oxide synthase (iNOS/NOS2A) contained an SXR-responsive element, and that increased expression of iNOS is a primary and direct response to SXR activation [52]. Production of iNOS increases the cellular levels of NO and RNS and a significant association between iNOS and p53 expression has been demonstrated at the mRNA and protein levels [55]. Accordingly, we examined expression of iNOS mRNA in MCF-7 and ZR-75-1 cells treated with SXR activators. MCF-7 cells were treated with SXR activators for 24, 48, and 72 hours, whereas ZR-75-1 cells were treated for 18 and 24 hours. These time points were selected because they precede those at which we observed up-regulation of p53 and its target genes in these cells, thus allowing the detection of prior events. We found that three different SXR activators increased iNOS mRNA levels in MCF-7 (Figure 4A) and ZR-75-1 cells (Figure 4B), suggesting that SXR activation, *per se*, and not other properties of the compounds was increasing iNOS mRNA levels. Increased iNOS expression was observed as early as 24 hours post-treatment in MCF-7 cells, whereas it could be detected starting at 18 hrs in ZR-75-1 cells. iNOS expression subsequently decreased in clotrimazole treated MCF-7 cells at 48 and 72 hours and in ZR-75-1 cells at 24 hrs. This is likely due to the reported negative regulation of the iNOS promoter by NO production and by activated p53 [56].

Under normal conditions, calmodulin (CaM) binding is necessary for the stabilization and activation of iNOS. When CaM levels are insufficient, newly synthesized iNOS is rapidly degraded through the calpain and proteasomal systems [57]. Therefore, we tested the expression of CaM mRNA in MCF-7 cells treated with rifampicin or solvent control for 3 hrs, 9 hrs, 12 hrs and 24 hrs and ZR75-1 cells treated with rifampicin or solvent control for 3 hrs, 9 hrs, 12 hrs and 18 hrs using QRT-PCR. We observed a transient up-regulation in steady state levels of CaM mRNA at 9 hrs in MCF-7 cells and at 18 hours in ZR75-1 cells (see Additional file 1: supplementary Figure 3A and 3B). To further confirm that this transient increase in CaM mRNA translated into sustained increase in protein level we tested the protein expression of CaM in MCF-7 and ZR-75-1 cells treated with rifampicin, anandamide or solvent control for 24 hrs and 18 hrs respectively. These timings correspond with when we noted up-regulation of iNOS mRNA in these cell lines. As shown in Figure 4C, CaM protein was up-regulated in comparison to the solvent control, DMSO by both SXR activators in both MCF-7 and ZR-75-1 cells. This is in accord with the notion that increased iNOS activity requires increased CaM to stabilize the newly synthesized iNOS [58]. In MCF-7 cells CaM mRNA is down by 24 hrs but CaM protein was still up till 24 hrs by both SXR activator compounds, rifampicin and anandamide suggesting long half-life of the protein.

Two approaches were employed to confirm that the elevated levels of iNOS correspond with increased NOS activity. First, we tested NOS activity in homogenates made from MCF-7 cells treated with 10 μM rifampicin or solvent control for 24 and 48 hours by measuring in-vitro biochemical conversion of ^14^C L-arginine into ^14^C L-citrulline as a direct measure for iNOS activity [59]. Second, we determined the NO concentration in cell free supernatant from SXR activator treated MCF-7 cells by measuring the total nitrate plus nitrite (the stable metabolites of NO) present in the media as an indirect measure of iNOS activity by the Greiss method [36]. Treatment of MCF-7 cells with rifampicin elicited a significant increase in NOS activity (Figure 4D). Treatment with a cytokine cocktail was used as a positive control for iNOS induction [60]. The increased NOS activity by either rifampicin or by cytokine cocktail treatment could be completely blocked by either a non-specific NOS inhibitor, L-N(G)-monomethylarginine (L-NMMA) [61] or the iNOS specific inhibitor 1400 W [62] (Figure 4D). Together, these results confirm that the increase in NOS activity in MCF7 cells resulting from treatment with SXR activators is primarily due to increased iNOS levels and activity.

Consistent with increased iNOS expression and NOS activity, the total nitric oxide level in the culture medium of MCF-7 cells treated with 10 μM rifampicin, anandamide, or clotrimazole for 48 hours were also increased (Figure 4E), confirming that iNOS-dependent increased production of NO could be one important mechanism through which SXR activators promote apoptosis in these cells. As expected based on the NOS activity, NO production was also blocked in SXR activated cells using the inhibitors 1400 W or L-NMMA (see Additional file 1: supplementary Figure 3C).

To assess whether SXR activation also affected the expression of eNOS (endothelial NOS), MCF-7 and ZR-75-1 cells were treated with 10 μM rifampicin for 24 and 18 hrs respectively. Treatment with SXR activators selectively increased the expression level of iNOS but not of eNOS in both breast cancer cell lines (see Additional file 1: supplementary Figure 3D). These results further support that observed increase in NOS activity and NO by SXR activators is primarily due to increased levels of iNOS.

Since increased NO levels have been reported to lead to p53 stabilization and activation, we next studied the requirement for NO in the up-regulation of p53 and subsequent cell cycle arrest and apoptosis that we observed in breast cancer cells. We treated MCF-7 cells with rifampicin in the presence or absence of L-NMMA or 1400 W, followed by QRT-PCR to measure the expression level of p53. Pre-treatment of MCF-7 cells with L-NMMA or 1400 W completely blocked the up-regulation of p53 observed following rifampicin treatment (Figure 4F). Therefore, we infer that increased cellular NO levels are required for up-regulation of p53 and its target genes in response to SXR activation.

Treatment with 1400 W itself activated p53 expression in breast cancer cells (Figure 4F). Since many known drugs activate SXR, we tested the ability of L-NMMA and 1400 W to activate SXR by measuring levels of the SXR target gene CYP3A4. Treatment with 1400 W, but not L-NMMA, increased CYP3A4 levels. We concluded that 1400 W activates SXR, which might explain its ability to up-regulate p53 (see Additional file 1: supplementary Figure 3E). Even though 1400 W can activate p53 on its own it was still able to inhibit rifampicin induced p53 up-regulation (Figure 4F). One possible explanation is that rifampicin is a higher affinity ligand for SXR than is 1400 W. Thus, in the absence of rifampicin, 1400 W can bind to and activate SXR. However, in the presence of rifampicin, the 1400 W is competed out and functions only as an iNOS inhibitor. Although this hypothesis needs further exploration, but since both of the NOS inhibitors (L-NMMA and 1400 W) were able to inhibit p53 up-regulation by SXR activators, we infer that that the SXR activator-induced increase in p53 expression is mediated through iNOS and the up-regulation of p53 by 1400 W does not affect our conclusion.

### Activity of SXR is sufficient to inhibit the growth of MCF-7 cells

**Figure 5:**
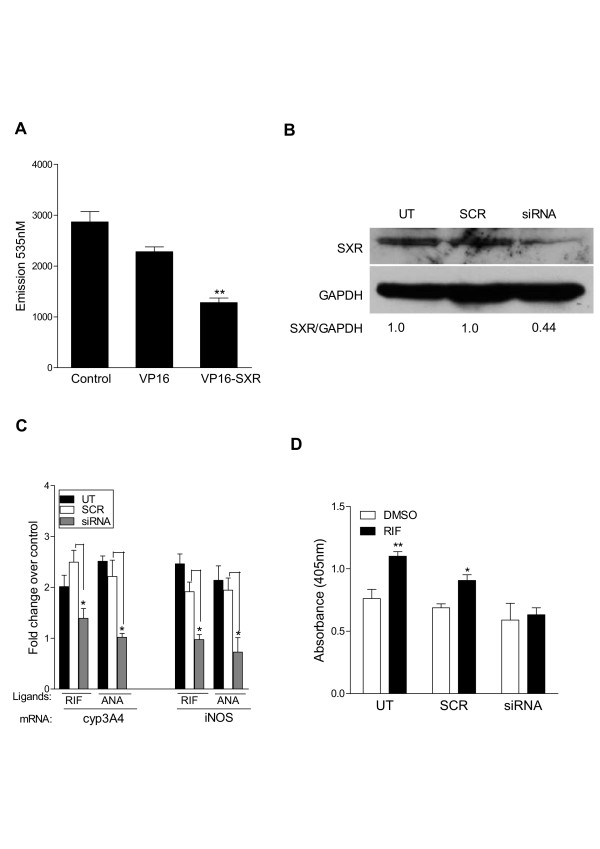
**Activated SXR is necessary and sufficient to inhibit the growth of breast cancer cells.** A) Constitutively active SXR is sufficient to decrease the growth of MCF-7 cells. MCF-7 cells were transfected with VP16 or VP16-SXR in the presence of pDSRed, and red fluorescent cells were sorted, plated and grown for an additional 4 days. Cell proliferation was measured by fluorescence assay as above and the experiment was repeated at least twice. ** represents P ≤ *0.01 *in comparison to VP-16 (by Student's t test). B) SXR expression is reduced by siRNA directed against SXR. MCF-7 cells were transfected with siRNA against SXR (siRNA) or scrambled siRNA (SCR) (100 nM each) for 72 hrs was tested for SXR protein expression using anti-412 SXR antibody. The chemiluminescent bands were analyzed by spot densitometry analysis using FluorChem AlphaEase FC software (Alpha Innotech) C) Ligand induced up-regulation of iNOS is directly regulated by SXR. SXR-siRNA but not SCR transfection blocked the ability of SXR ligands (RIF and ANA) to induce up-regulation of CYP3A4 and iNOS. Un-transfected MCF-7 cells, or cells transfected with siRNA or SCR sequence (36 nM each) were treated with 10 μM RIF or ANA for 48 hr. Target gene expression was tested by QRT-PCR. The data are shown as average fold induction relative to solvent control in triplicates ± S.E.M. * represents P ≤ *.05 *in comparison to SCR (by student's t test). D) SXR activation induced apoptosis is blocked by SXR siRNA. Apoptosis was measured in equal number of MCF-7 cells induced with SXR activator rifampicin or solvent control DMSO for 72 hours after knocking down SXR expression using SXR specific siRNA. The cells transfected with a non-specific scrambled siRNA or un-transfected cells were used as a control. The experiment was repeated twice in triplicates. The absorbance at 405 nM represents apoptotic cells and data are shown as mean absorbance in triplicates ± S.E.M. * represents *P *≤ *.05 *in comparison to DMSO solvent control.

Although SXR activators were able to inhibit breast cancer cell proliferation, the compounds tested can potentially act through other pathways to stop cancer cell growth. To establish whether SXR activation, *per se*, was responsible for the observed effects on cell proliferation, we employed a constitutively active form of SXR [63]. This construct, VP16-SXR, contains the strong transcriptional activation domain from the herpes simplex virus protein VP16 fused to the amino terminus of full length SXR-1. VP16-SXR is transcriptionally active in the absence of added ligands [63]. Plasmids expressing VP16-SXR and pDSRed (which expresses a red fluorescent protein) were transiently transfected into MCF-7 cells. Red fluorescing cells (which are assumed to contain both VP16-SXR and pDSRed) were separated from non-fluorescing cells using fluorescence-activated cell sorting. The sorted red fluorescent cells were cultured for an additional four days, then harvested for cell proliferation assays. Proliferation of VP16-SXR-transfected cells was strongly inhibited compared with cells transfected with empty plasmid, or with VP16 alone (Figure 5A). Therefore, we infer that SXR activation itself decreased the proliferation of breast cancer cells, supporting our model that the test compounds also exerted their effects on proliferation through SXR.

We further confirmed our model by testing the gene expression level of iNOS and p53 and p53 dependent target genes in VP16 or VP16-SXR transfected MCF-7 cells. The expression of iNOS and p53 and p53 dependent genes was strongly induced in VP16-SXR transfected cells in comparison to VP16 alone transfected cells (see Additional file 1: supplementary Figure 4A). This further supports our model that the SXR activator-dependent effects on proliferation of breast cancer cells are mediated specifically through SXR-induced increase in iNOS and p53 and p53 dependent genes and not because of other properties of the compounds.

### SXR knockdown by siRNA inhibits the increased expression of iNOS

As a final confirmation of the requirement for SXR in the regulation of iNOS expression and iNOS-mediated up-regulation of p53 and its target genes in breast cancer cells, we investigated the effects of SXR loss-of-function on the induction of gene expression by SXR ligands. MCF-7 cells were transfected with scrambled or SXR specific siRNA [27,37]. Consistent with previously published reports treatment with a specific siRNA duplex against SXR, but not with a control scrambled siRNA, reduced the SXR mRNA level by more than 80% (see Additional file 1: supplementary Figure 4B), and protein levels by more than 50% in MCF-7 cells (Figure 5B). Rifampicin and anandamide induced up-regulation of both CYP3A4 and iNOS mRNA expression was also significantly inhibited by the SXR siRNA in these cells (Figure 5C). Similarly the SXR activator induced expression of p53 and p21 was also significantly inhibited by siRNA (see Additional file 1: supplementary Figure 4C).

As a final confirmation of the functional impact of SXR knockdown, we measured apoptosis in SXR siRNA treated cells. MCF-7 cells were transfected with scrambled or SXR specific siRNA. 36 hours after transfection, the cells were treated with rifampicin or solvent control. 72 hr post-treatment apoptosis was measured in equal number of cells using cell death detection ELISA. As shown in Figure 5D the un-transfected and SCR transfected cells showed a significant increase in apoptosis after rifampicin treatment compared with controls. In contrast, there was no induction of apoptosis by rifampicin in MCF-7 cells transfected with SXR-siRNA (Figure 5D). These results confirm the requirement for SXR function to mediate the iNOS and p53-dependent downstream pathways resulting in apoptosis and cell cycle arrest described above.

### Knockdown of p53 inhibits up-regulation of p21 and BAX

**Figure 6:**
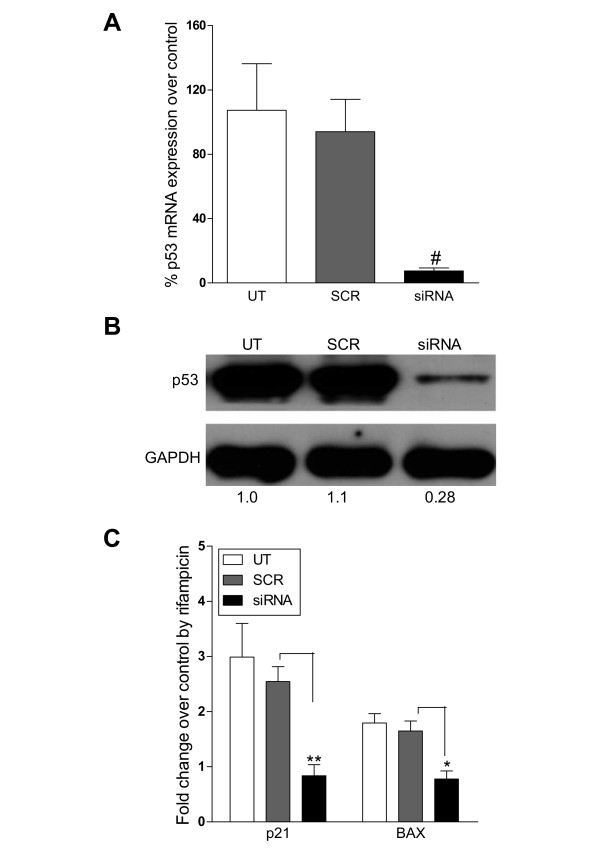
**SXR induced apoptosis is p53 dependent**. A) MCF-7 cells were transiently transfected with siRNA specific against p53 (siRNA) or scrambled siRNA (SCR) (50 nM each). The knockdown efficiency was measured by measuring mRNA (A) or protein levels of p53 (B) after 48 and 72 hours of transfection respectively. The protein bands were analyzed by FluorChem AlphaEase FC software (Alpha Innotech).*# represents P *≤ *.001 *in comparison to SCR (by student's t test) C) SXR induced up-regulation of cell cycle regulatory and apoptotic gene is dependent on p53. MCF-7 cells un-transfected (UT) or transfected with SCR or p53 specific siRNA (siRNA) were induced with SXR activator, rifampicin or DMSO control for 72 hours. The mRNA expression level of p21 and BAX was measured by QRT-PCR. The data are shown as average fold induction relative to DMSO control in triplicates ± S.E.M. * represents P ≤ .05 and ** represents P ≤ *.01 *in comparison to SCR (by student's t test).

To further confirm that activation of SXR and its subsequent anti-proliferative effects in breast cancer cells are mediated through a p53 dependent mechanism, we knocked down p53 using siRNA. Transient transfection of MCF-7 cells with p53 specific siRNA (siRNA) but not with a scrambled siRNA (SCR) reduced p53 mRNA level by more than 90% (Figure 6A) and camptothecin stabilized p53 protein level by more than 70% (Figure 6B). As predicted, induction of p21 and BAX mRNA by rifampicin is significantly blocked in p53 siRNA transfected, but not in SCR siRNA transfected cells (Figure 6C). These results confirm the requirement of p53 for the SXR-mediated growth inhibitory and apoptotic effects on breast cancer cells.

## Discussion

The orphan nuclear receptor SXR is known to regulate the expression of target genes involved in all three phases of steroid and xenobiotic metabolism in the liver and gut. However, SXR is also expressed in bone, kidney, lung, endometrium and breast tissue [22,24-26,39]. Most of these tissues do not contribute significantly to xenobiotic metabolism; hence, SXR may be performing other functions. Here we have defined a new mechanism by which SXR transcriptional activation affects the growth of breast cancer cells. Activation of SXR in ER^+^p53^wt ^breast cancer cells inhibits their proliferation through a cascade of events beginning withinduction of iNOS and production of NO that ultimately leads to cell cycle arrest and apoptosis.

**Figure 7:**
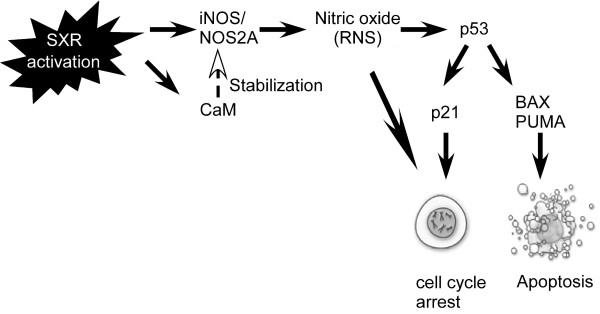
**Model depicting cell cycle arrest and apoptosis in breast cancer cells associated with SXR activation**. SXR activation by xenobiotic ligands causes coordinated up-regulation of iNOS and its protein activator calmodulin (CaM); CaM binding to iNOS promotes the oxidative burst and bacterial killing or apoptosis and necrosis. Following activation of iNOS the increased NO levels cause stabilization of p53 protein and subsequent up-regulation of p53 mRNA [49,55]. p53 up-regulates cell-cycle regulatory (p21) and apoptotic genes (BAX and PUMA). The increase in p21 causes G1 arrest in cells. Ultimately, the unchecked increase in the levels of the pro-apoptotic genes BAX and PUMA commits the cell to undergo apoptosis. Closed arrows indicate genes induced or up-regulated. The open arrow indicates that CaM stabilizes iNOS, facilitating its enzymatic activity.

Our experiments demonstrated increased steady state levels of iNOS mRNA in both MCF-7 and ZR75-1 cells, as well as increased iNOS activity accompanied by accumulation of NO in MCF-7 cells treated with SXR activators. We also observed increase in CaM mRNA and protein levels following SXR activation in both MCF-7 and ZR-75-1 cells. Consistent with this observation, increased CaM is expected to accompany induced iNOS expression since CaM binds to, and is required for activity of newly synthesized iNOS [57]. Increases in NO (48 hour post-treatment) levels were followed by increased expression of p53, and p53 target genes such as p21, BAX and PUMA (72 hour post-treatment), which finally led to apoptosis and G1 arrest (Figure 7) in MCF-7 cells. Other reports demonstrated increased stability and accumulation of p53 by NO [56] and a strong association of p53 expression with iNOS expression [55]. Previous work has also established that accumulated p53 leads to up-regulation of cell cycle regulatory proteins, and pro-apoptotic proteins such as p21 and BAX [50,51] and induction of these genes leads to G0/G1 arrest [50] and apoptosis [46,47,50]. Therefore, the mechanism we have identified links SXR activation with a well-characterized pathway capable of mediating cell proliferation and apoptosis. Loss-of-function and gain-of-function studies shown above demonstrated that SXR activation is necessary and sufficient for this pathway. Therefore, we infer that the regulation of cell proliferation and apoptosis in response to xenobiotic ligands in breast cancer cells is a novel cellular function for SXR that requires further exploration.

It was previously established that RNS/NO cause p53 stabilization and that the stabilized p53 is transcriptionally active [51]. NO-induced p53 stabilization is hypothesized to result from phosphorylation at serine-15 of p53 [49]. This phosphorylated form of p53 has been associated with attenuated p53 nuclear export [64]. In turn, nuclear levels of activated p53 are subject to negative regulation by Mdm2, which functionally inactivates p53, facilitates p53 ubiquitination, nuclear export, and proteasomal degradation. Whether or not phosphorylation at serine 15 disrupts the p53/Mdm2 interaction is currently controversial [65]. However, it is clear that blocking p53 nuclear export stabilizes active p53 [66] and that NO exposure leads to nuclear p53 accumulation [56]. Moreover, expression of the iNOS gene has been shown to have a strong association with p53 expression at both mRNA and protein levels [55]. Increased BAX, PUMA and p53 mRNA levels were not observed by all three SXR activators until 72 hours in MCF-7 cells, whereas iNOS was up-regulated as early as 24 hrs after treatment with SXR activators in our experiments (Figure 3A and Figure 4A). This timing supports our model that the primary effect of SXR activation in these breast cancer cells is to increase steady-state levels of iNOS mRNA which then leads to increased production of NO. Increased NO levels up-regulate expression of p53. The elevated levels of p53 further up-regulate expression of p21 and that of the pro-apoptotic p53 target genes BAX and PUMA in breast cancer cells (Figure 7). Inhibition of NOS blocked the induction of p53 and knock down of p53 inhibited the induction of p21 and BAX in response to SXR activators. Both of these results further confirmed our model that p53 is acting down-stream of NOS and is required for up-regulating the expression of cell cycle regulatory and pro-apoptotic genes such as p21, BAX and PUMA in breast cancer cells in response to SXR activation.

Interestingly, expression of the cell cycle regulatory gene p21(Waf1/Cip1) can also be induced by NO [50,67]. Moreover, nitric oxide can have cytostatic effects on cells by directly influencing the cyclin D1 expression, independent of p53 [54]. In our experiments MCF-7 accumulated in G1/S phase of cell cycle starting as early as 24 hours post-treatment. In contrast, p53 and its target genes did not started going up until 48 hours, indicating p53 independent cytostatic effects of nitric oxide at earlier time points.

Experiments in p53 null mice as well as in p53^wt ^versus p53^mut ^lymphoblastoid cells have demonstrated that NO induces apoptosis, and that this effect is dependent upon the presence of wild type p53 [68]. We detected elevated NO and apoptosis in MCF-7 cells treated with SXR activators, and increases in iNOS mRNA as well as mRNAs encoding p53 and p53-target genes in MCF-7 and ZR75-1 cell lines. Both of these cell lines are p53 wild type; therefore, our results are consistent with the earlier findings. As expected, we did not observe the induction of p53 target genes by SXR activators in cells transfected with p53 siRNA which again emphasize the role of p53 in our model.

Previously published reports have suggested that there is an inverse relationship between ER and SXR levels in breast and endometrial tissues [22,25]. Both of the cell lines tested in this study are ER positive. However, in our preliminary experiments with ER^+ ^and ER^- ^breast cancer cell lines and consistent with previously published reports with breast cancer cell lines [25], we did not observe an inverse relationship between ER and SXR protein levels (data not shown). It is possible that this may be because insufficient samples have been examined and this point requires further exploration with bigger sample size. It would also be of interest to see if the anti-proliferative effects of SXR are limited to ER^+ ^breast cancer cells.

Interestingly, in this study we also found that although MCF-7 and ZR-75-1 cells express almost equal amount of SXR protein (Figure 1), the MCF-7 cells were considerably more sensitive in anti-proliferative response to SXR activators in comparison to ZR-75-1 cells (Figure 2A). There can be many reasons for differences in the sensitivity of these cells towards SXR activators. One plausible and likely explanation is that ZR-75-1 cells express much lower levels of p53 in comparison to MCF-7 cells http://www.mdanderson.org/departments/cancerbiology/dIndex.cfm?pn=31062032-B0EB-11D4-80FB00508B603A14. The topoisomerase I inhibitor, camptothecin can only induce a ~3 fold increase in p53 protein levels in ZR75-1 cells compared with ~18 fold increase in MCF-7 cells (please compare figure 3C and Figure 3D). This is consistent with the possibility that differences in the sensitivity of these cells to SXR activators is more closely related to the inducibility of p53, than to the levels of SXR.

A very recent study has shown that SXR activators increased the expression of organic anion transporter, enhancing estrogenic effects in breast cancer [69]. The differences in the pro-proliferative vs. pro-apoptotic effects of SXR in this study vs. our study can have several possible causes. There was a different experimental design in that the other study grew the breast cancer cells in estrogenic conditions vs. our experiments in which the cells were grown in estrogen depleted conditions (stripped medium, phenol red free) or the choice of breast cancer cell lines (MCF-7 and ZR-75-1 vs. T47-D). In addition, MCF-7 cells express much lower level of OATP1a2 than T47-D and T47-D are p53^mut^.

Recent studies have reported anti-apoptotic effects of SXR in liver and colon cancer cells [29,34] and proliferative effects in ovarian cancer cells *in-vitro *[70], whereas SXR activation, *in-vivo*, has been suggested to have pro-apoptotic effects in colon tissues [33]. Here we have shown that activation of SXR is pro-apoptotic in breast cancer cells. The ability of SXR to be pro-apoptotic in one tissue and anti-apoptotic in others may result from cell-type specific effects of SXR, or of SXR-induced NO. We previously, showed that SXR can regulate gene expression in a tissue specific manner based on the levels of co-repressor NCoR expressed in these tissues [71]. Therefore, we propose that SXR activation might activate a different panel of gene in liver or colon tissue in comparison to breast tissue which may explain its different role between tissues. Moreover, the association between RNS/NO and apoptosis is also very complex. RNS/NO can provoke apoptosis or alternatively antagonize cell death pathways, depending on the cell type, NO concentration, redox state of the cell, pro-versus anti-apoptotic balance of an individual cell, and the subcellular localization of NO synthesis (reviewed in [53]). Therefore, the threshold levels for RNS to trigger apoptosis will likely differ from one cell to another. Interestingly, in accordance with our results different groups have reported induction of NO by many different SXR activators such as rifampicin, tamoxifen and anandamide in different cell types [72-74]. These studies from different groups suggest that SXR might commonly activate NO in different cells but depending on the cellular milieu, SXR generated NO can be pro-apoptotic or anti-apoptotic. Taken together, these studies suggest that SXR may have cell type specific effects on proliferation/apoptosis pathways, particularly in cancer cells.

These results also raise an intriguing question – is SXR expression pro- or anti-proliferative in breast cancers, *in vivo? *Understanding whether SXR is pro- or anti-proliferative in breast cancers is very important for optimizing breast cancer therapies because many commonly used chemotherapeutic agents (tamoxifen, taxol, cyclophosphamide, cisplatin) are SXR activators. The novel link we have established between SXR and breast cancer requires further investigation using appropriate *in vivo *systems. If, as we have shown, SXR activation is anti-proliferative, then therapies or preventive measures that target SXR without inducing their own metabolism will provide an important adjunct to current therapies. If SXR expression promotes, rather than inhibits the growth of cancer cells, then treatment with drugs that activate SXR will ultimately be counterproductive to tumor treatment. In this event, treatment of patients with standard therapies together with SXR antagonists [28,75], or with SXR-transparent chemotherapeutic agents could prove beneficial both in blocking tumor growth and in improving the therapeutic efficacy of existing agents.

## Conclusion

Taken together, the data presented above show that activation of SXR induces apoptosis in p53^wt ^breast cancer cells that is mechanistically dependent upon induction of iNOS and NO-induced accumulation of p53 in cells. Our results have established a novel link between SXR and p53 induction and apoptosis in breast cancer cells. There have been scattered reports on xenobiotic metabolism and breast cancer. However, it was not clear what pathways or genetic factors are involved. Our results represent the first identification of an association between a well established xenobiotic receptor, SXR and apoptosis of breast cancer cells. Further in depth studies providing mechanistic insights into the role of SXR in proliferation and apoptosis of mammary epithelial cells *in-vivo *will have significant impact on breast cancer.

## Abbreviations

iNOS/NOS2A: Inducible Nitric Oxide Synthase; NO: Nitric Oxide, QRT-PCR: Quantitative Real Time RT-PCR; ELISA: Enzyme-Linked ImmunoSorbent Assay; ROS: Reactive Oxygen Species; RNS: Reactive Nitrogen Species; eNOS: Endothelial NOS; CaM: Calmodulin; L-NMMA: L-N(G)-MonoMethylArginine.

## Competing interests

BB is a named inventor on patents related to SXR, held by the Salk Institute for Biological Studies and licensed to for-profit entities. MT and SV declare no competing interests.

## Authors' contributions

SV carried out the proliferation, flow cytometery and apoptosis ELISA. SV also carried out the gene expression, protein analysis and siRNA experiments and performed the statistical analysis. MT participated in the design of the study, proliferation assay and carried out the constitutively active SXR (VP16-SXR) experiments. SV and MT equally contributed in drafting the manuscript. BB conceived of the study, and participated in its design and coordination and helped to draft the manuscript. All authors read and approved the final manuscript.

## Pre-publication history

The pre-publication history for this paper can be accessed here:

http://www.biomedcentral.com/1471-2407/9/3/prepub

## Supplementary Material

Additional file 1 Supplementary data. Click here for file
