# Supplementary material for: Activation of the steroid and xenobiotic receptor, SXR, induces apoptosis in breast cancer cells
Source: BMC Cancer. 2009 Jan 5;9:3. doi: 10.1186/1471-2407-9-3 (PMC2631587; doi:10.1186/1471-2407-9-3)
Supplement: Additional file 1 — Supplementary data. [file 1471-2407-9-3-S1.doc]

**Supplementary data:**

Figure S1. Validation of the specificity of the polyclonal SXR antibody anti-412

SXR antibody, anti-412 detects SXR/PXR band in liver and intestine tissues from SXR/PXR wild type (WT) mice but not in corresponding tissues from SXR/PXR knock out (KO) mice. Equal amounts of cell lysates made from liver and intestine tissues of SXR/PXR WT and KO mice were subjected to western blot analysis using anti-412 antibody. The band of expected size ~50KD size is present in tissues from WT mice but not in tissues from KO mice. Note that the non-specific band above 37KD size marker did not change between WT and KO mice.

Figure S2: The ability of a concentration series of compounds to activate SXR was determined in transfection assay.

COS-7 cells transiently transfected with Gal-SXR and tk(MH-100)_4_-luc plasmid were treated with a concentration series of test compounds that can activate SXR. Rutin was used as a negative control compound. Data represents mean of triplicate values ± S.E.M.

Figure S3: SXR and NO signaling.

SXR activation causes transient and rapid increase in steady state levels of calmodulin mRNA. Total RNA extracted from A) MCF-7 and B) ZR-75-1 cells treated with or without rifampicin (10 μM) for indicated time points were tested for calmodulin expression by QRT-PCR. *** represents P* ≤*.01* compared to control and * *represents P≤.05* compared to control (by student’s t test) . C) The NOS inhibitors L-NMMA and 1400W inhibit rifampicin induced increase in nitric oxide levels. MCF-7 cells were pre-treated with or with-out 1400W (10μM) or L-NMMA (500μM) for 1 hour and then stimulated by rifampicin (RIF) for 24 hours. Nitric oxide levels were determined in the cell free culture supernatant by Griess method. The experiment was replicated in duplicates in independent experiments. D) Activation of SXR specifically activates iNOS expression in breast cancer cells. QRT-PCR was performed on total RNA isolated from MCF-7 and ZR-75-1 cells treated with rifampicin 10μM for 24 and 18 hrs respectively using iNOS and eNOS primers. nNOS expression was not detected. The data is represented as fold induction over control in triplicates ± S.E.M. E) 1400W but not L-NMMA activates SXR. MCF-7 cells growing in culture were treated with rifampicin, 1400W or L- NMMA. QRT-PCR was performed on total RNA isolated from cells after 72 hrs of treatment using cyp3a4 primers. The data is shown as average fold induction relative to solvent control in triplicates ± S.E.M. These results were replicated in at least two independent experiments.

**Figure S4: Effects of SXR on p53 and its target genes**

**A)** Constitutively active SXR increases the expression of iNOS and p53 and p53 dependent target genes in breast cancer cells. MCF-7 cells were transfected with plasmids expressing a constitutively active variant of SXR (VP16-SXR) or VP16 alone. QRT-PCR was performed on total RNA isolated from cells 24 hr post-transfection using iNOS, p53, p21, BAX and PUMA primers. Cyp3A4 was used as a positive control for SXR activation. The data is shown as average fold induction relative to VP16 control in triplicates ± S.E.M. **B)** SXR expression is reduced by siRNA directed against SXR. MCF-7 cells were transfected with siRNA against SXR (siRNA) or scrambled siRNA (SCR) (100 nM each) for 48 hrs and tested for SXR mRNA expression using Q-RTPCR.  *# represents P* ≤*.001* in comparison to SCR (by student’s t test). **C)** Ligand induced up-regulation of p53 and p21 is directly regulated by SXR. SXR-siRNA but not SCR transfection blocked the ability of SXR ligands (RIF and ANA) to induce up-regulation of p53 and p21. Un-transfected MCF-7 cells, or cells transfected with siRNA or SCR sequence (36 nM each) were treated with 10 μM RIF or ANA for 48 hr. Target gene expression was tested by QRT-PCR. The data are shown as average fold induction relative to solvent control in triplicates ± S.E.M. *** represents P* **≤***.01 and # represents P* **≤***.001* in comparison to SCR (by student’s t test).
